# Caffeine increases sugar-sweetened beverage consumption in a free-living population: a randomised controlled trial

**DOI:** 10.1017/S000711451400378X

**Published:** 2015-01-28

**Authors:** Russell S. J. Keast, Boyd A. Swinburn, Dhoungsiri Sayompark, Susie Whitelock, Lynn J. Riddell

**Affiliations:** 1 Centre for Physical Activity and Nutrition Research, School of Exercise and Nutrition Sciences, Deakin University, 221 Burwood Highway, Burwood, VIC3125, Australia; 2 WHO Collaborating Centre for Obesity Prevention, Deakin University, 221 Burwood Highway, Burwood, VIC3125, Australia; 3 School of Population Health, University of Auckland, Auckland, New Zealand; 4 Faculty of Science and Technology, Rajamangala University of Technology Tawan-ok, Chonburi, Thailand

**Keywords:** Sugar-sweetened beverages, Caffeine, Free-living populations

## Abstract

Excessive sugar-sweetened beverage (SSB) consumption has been associated with overweight and obesity. Caffeine is a common additive to SSB, and through dependence effects, it has the potential to promote the consumption of caffeine-containing foods. The objective of the present study was to assess the influence that caffeine has on the consumption of SSB. Participants (*n* 99) were blindly assigned to either a caffeinated SSB (C-SSB) or a non-caffeinated SSB (NC-SSB) group. Following randomisation, all participants completed a 9 d flavour-conditioning paradigm. They then completed a 28 d *ad libitum* intake intervention where they consumed as much or as little of C-SSB or NC-SSB as desired. The amount consumed (ml) was recorded daily, 4 d diet diaries were collected and liking of SSB was assessed at the start and end of the intervention. Participants (*n* 50) consuming the C-SSB had a daily SSB intake of 419 (sd 298) ml (785 (sd 559) kJ/d) over the 28 d intervention, significantly more than participants (*n* 49) consuming the NC-SSB (273 (sd 278) ml/d, 512 (sd 521) kJ/d) (*P*< 0·001). A trained flavour panel (*n* 30) found no difference in flavour between the C-SSB and NC-SSB (*P*>0·05). However, participants who consumed the C-SSB liked the SSB more than those who consumed the NC-SSB (6·3 *v.* 6·0 on a nine-point hedonic scale, *P*= 0·022). The addition of low concentrations of caffeine to the SSB significantly increases the consumption of the SSB. Regulating caffeine as a food additive may be an effective strategy to decrease the consumption of nutrient-poor high-energy foods and beverages.

Sugar-sweetened beverages (SSB) are micronutrient-void, energy-containing, readily available beverages that are consumed in large quantities around the world, with Americans being particularly high consumers^(^ ^1^ ^)^. Regular SSB consumers have higher energy intakes (up to 10 %) than non-consumers^(^ ^2^ ^–^ ^4^ ^)^, and, overall, there is supportive evidence associating overconsumption of SSB with a higher energy intake and the development of overweight and obesity^(^ ^5^ ^–^ ^7^ ^)^.

Based on industry reports, we have previously estimated that 63·4 % of SSB consumption was from caffeinated SSB (C-SSB)^(^ ^1^ ^,^ ^8^ ^)^. Manufacturers claim that caffeine is added as a flavour enhancer in SSB^(^ ^9^ ^)^; however, any flavour effect of caffeine will be a function of its concentration^(^ ^10^ ^,^ ^11^ ^)^, and we and other researchers^(^ ^8^ ^,^ ^10^ ^,^ ^12^ ^,^ ^13^ ^)^ have questioned whether caffeine at concentrations found commonly in SSB, has any flavour activity. Despite the limited evidence for a role in flavour, the amount of caffeine delivered in 500 ml common cola soft drinks (approximately 53–65 mg, 0·55–0·67 mm) is high enough to modify consumption behaviour, potentially increasing the consumption of the beverage as a form of caffeine redosing^(^ ^14^ ^)^. Indeed, studies have shown that the consumption of caffeine promotes a dependence that is reinforced with repeat consumption of caffeine-containing beverages^(^ ^15^ ^–^ ^17^ ^)^.

Caffeine may promote the consumption of these beverages via the development of flavour preferences where individuals associate (unconsciously) a food/flavour with its ability to alleviate caffeine-withdrawal symptoms^(^ ^18^ ^–^ ^20^ ^)^. Flavour preference for sweetness is immediate, whereas the influence of caffeine occurs post-ingestion^(^ ^21^ ^)^. These post-ingestive effects (increased vigilance and attention, enhanced mood and arousal, as well as enhanced motor activity^(^ ^22^ ^)^) are cyclic and are replaced by withdrawal symptoms if caffeine is not redosed^(^ ^18^ ^)^. When caffeine is redosed, the reversal of caffeine-withdrawal symptoms increases the preference for the food/beverage. To date, no studies have shown that caffeination of a beverage increases its consumption in a free-living population, although laboratory-based studies have shown that caffeine-deprived participants increase the liking and consumption of caffeinated beverages^(^ ^18^ ^,^ ^19^ ^)^. Anecdotal evidence also suggests that caffeine may have a role in increasing consumption as there are an increasing number of non-traditional high-energy caffeine sources such as candies, ice creams, breakfast cereals, yogurt and chewing gums entering the food supply^(^ ^23^ ^)^. Redosing with caffeine becomes a problem when the form of redosing is high in energy, promoting increased energy intake.

The objective of the present study was to assess in a free-living population whether caffeine, at levels found in common cola SSB, increased SSB consumption compared with a flavour-equivalent non-caffeinated SSB.

## Methods

### Study design

The present study was a double-blind 6-week dietary intervention study. Participants completed a 4 d diet diary, SSB liking, and body weight and height measurements at the start and end of the intervention. Participants were masked as to the true purpose of the study, being told that it will be testing the palatability and liking of a lemon-flavoured SSB. Participants were randomly assigned to either a C-SSB (0·57 mm-caffeine) or a non-caffeinated SSB (NC-SSB) group. Participants were asked to consume 600 ml of the assigned SSB per d in a 9 d flavour-conditioning run-in phase before a 28 d *ad libitum* intake phase. SSB were delivered weekly to all the participants and empty bottles returned, and participants recorded SSB consumption daily during the study. Consumption of the SSB (in ml) was the primary outcome measure. At the end of the study, the true nature of the study was disclosed to the study participants who were informed that two beverages were used in the study, one non-caffeinated and one containing levels of caffeine comparable to commercially available cola beverages. Participants were then asked to identify whether they thought they were consuming the NC-SSB or C-SSB during the study.

An additional thirty participants who were part of descriptive flavour research programme at Deakin University assessed flavour difference between the two study beverages.

### Participants

Participants (*n* 123) were recruited from the area around Deakin University and Box Hill Institute campus, Melbourne, Australia between January and August 2010. Participants were eligible to participate if they were aged between 18 and 30 years, in good health, not pregnant or lactating, not using medications known to affect food intake or appetite, and weight stable (no change in body weight > ± 5 kg) in the last 6 months. All the participants were regular consumers of SSB (at least one SSB/week) and caffeine (tea, coffee and cola beverages daily). Participants were randomised into either the C-SSB or NC-SSB group using a computer-generated randomisation programme stratified by sex. Participants and research personnel involved with participant interaction were blinded to group assignment. The number of participants recruited was based on the observed variance in SSB consumption in a large nationally representative sample of 18-year-old Americans^(^ ^4^ ^)^; 100 participants were required to complete the study to give an 80 % chance of observing a 150 ml/d difference in intake between the C-SSB and NC-SSB. Ethical approval to conduct the study was obtained from the Deakin University Human Research Ethics Committee, and all participants provided informed written consent before participation. The present trial was registered at the Australian New Zealand Clinical Trials Registry (ACTRN12608000151336; http://www.anzctr.org.au).

### Experimental sugar-sweetened beverages

Carbonated soft drinks were manufactured specifically for the present study by Saxbys Soft Drinks. The nutrient composition (g/100 g), determined by chemical analysis, of the SSB was as follows: carbohydrate total 11·5 % and sugars 10·7 %; water 88 %. The C-SSB additionally contained 110 mg caffeine/l (57 mm-caffeine), which is equivalent to the concentrations of normal carbonated cola drinks.

### Flavour conditioning

All the participants were involved in the 9 d flavour-conditioning phase before the start of the intervention. Participants were allocated either the C-SSB or NC-SSB and instructed to consume one bottle (600 ml) per d for 9 d. This allowed the participants in the C-SSB condition to associate the flavour of the SSB with caffeine.

### Sugar-sweetened beverage intervention

SSB and dietary consumption was monitored during the intervention via the collection of diet dairies. A research dietitian explained how to accurately complete the SSB and 4 d diet dairies to all the participants. Participants would open a new bottle (600 ml) of SSB each day and record the volume of SSB consumed during the day by assessing the number of bottles and the volume remaining in the SSB container at the end of each day. For the 4 d food diaries, participants were asked to, where possible, weigh the foods they consumed, or use measuring cups, spoons or common serving sizes (e.g. one slice of bread), and to be specific, such as reporting the brand of food consumed, type of food (e.g. white or wholemeal bread), whether fat was added (e.g. oil or butter) and the cooking methods (e.g. baking, frying, steaming). If the food consumed was from a recipe, the subject was asked to include the recipe with the record and to state how much of it they consumed (e.g. whole, half). Participants were also supplied with the Otago University Booklet of Diet Assessment Photos to aid them in supplying measurable information on portion sizes. Diet diaries were completed over three weekdays and one weekend day. Participants were asked not to consume any other SSB during the intervention period and were instructed that the study SSB were only for their individual consumption. SSB were delivered weekly to the participants' home or collected from Deakin University, whichever was more convenient. Weekly SSB consumption records were collected at the time of SSB delivery, and were checked by a dietitian for accuracy; any questions raised by the participants could be answered at the time. Foods and the amount of food consumed were entered into Food Works 2009 (Xyris Software) and analysed using the AUSNUT 2007 database for foods, brands and supplements.

### Liking of sugar-sweetened beverages

Liking of the SSB was assessed at the start and end of the study. Participants were given a 30 ml sample of the C-SSB or NC-SSB, depending on their study randomisation, and asked to consume the volume and then rate their liking on a nine-point liking scale.

### Body composition

BMI (weight (kg)/height (m)^2^) was calculated from measured height and weight. Height, without shoes, was measured in duplicate to the nearest 0·1 cm with a wall-mounted stadiometer (Holtain Limited). Weight, in light clothing, was measured in duplicate to the nearest 0·1 kg on medical scales (model 708; Seca).

### Sugar-sweetened beverage difference test

A trained taste panel (*n* 30) was used to investigate any perceived flavour differences between the two study beverages using a tetrad sorting method. To this end, four 30 ml plastic medicine cups (McFarlane Medical and Scientific) containing 20 ml SSB (two caffeinated and two non-caffeinated), labelled with a three-digit code, were served in a random order across the participants. Participants were required to group the two samples they believed were the same (two groups of two) or guess the groupings if they did not know. The tetrad test was completed in triplicate by each participant. Participants were provided with water for rinsing between the samples. All testing was conducted in a specialised sensory-testing facility comprising seven individual computerised booths. Results were collected using Compusense five software (Compusense, Inc.). Each subject was isolated from other participants by vertical dividers, and there was no interaction between the participants.

### Statistical analysis

SPSS version 21.0 software (IBM SPSS) was used for the statistical analysis of the data. Numerical data are expressed as means and standard errors. Descriptive statistics were employed to describe demographic information, SSB consumption and dietary intake. Between-group differences from day 0 to 28 were analysed by two-way between-group ANOVA. Pearson's product–moment coefficient correlations were conducted between continuous variables to analyse the relationship between average SSB consumption, liking of SSB, BMI, and dietary intake. Results were considered to be statistically significant when *P*< 0·05.

## Results

### Participants

**Fig. 1:**
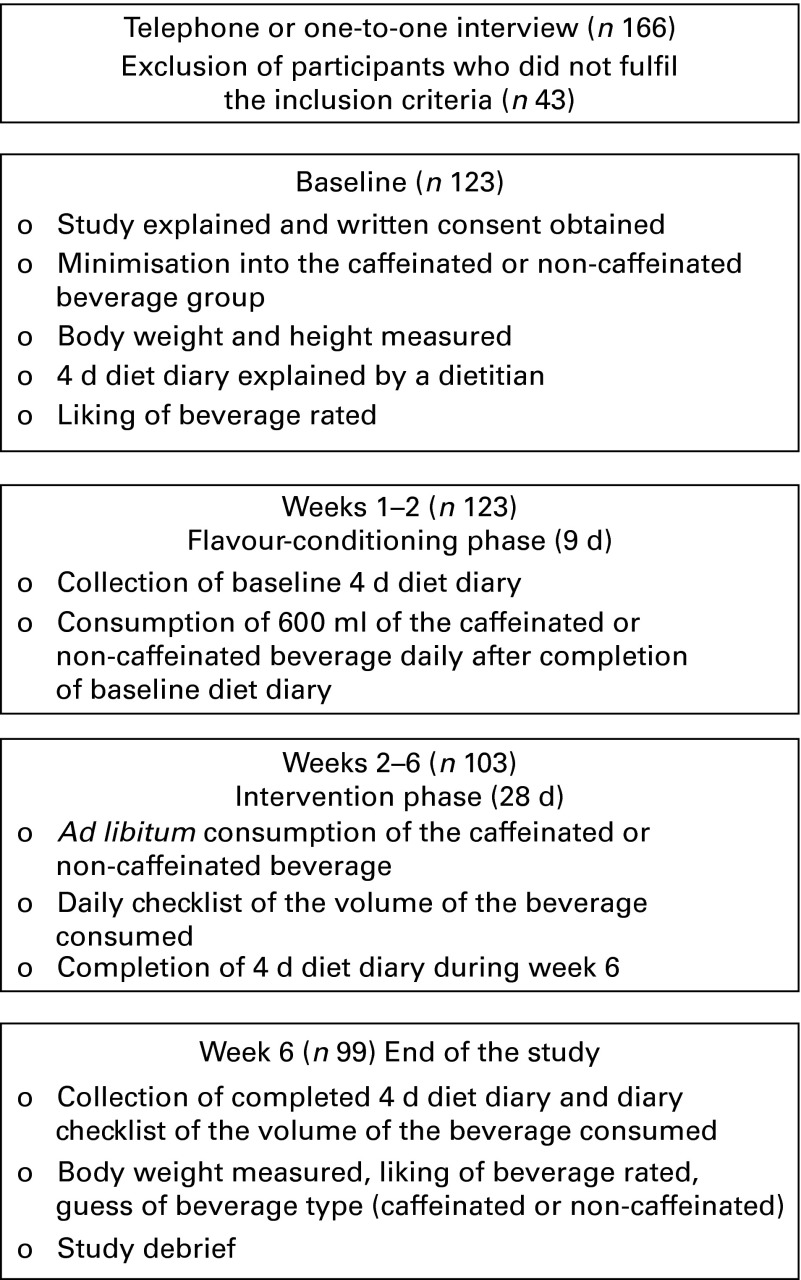
Flow chart of the study outline.

A total of 123 participants (49 % female, age 23 (sd 3) years) enrolled in the present study (Fig. 1). During week 1, twenty participants withdrew due to time pressures or a lack of response to research personnel, and during the intervention, a further four participants withdrew due to failure to respond to contact from research personnel. Finally, ninety-nine participants completed the study: *n* 49 in the NC-SSB group (53 % female, age 23 (sd 4) years and BMI 23·1 (sd 3·1) kg/m^2^) and *n* 50 in the C-SSB group (48 % female, age 22·5 (sd 2·8) years and BMI 22·9 (sd 3·4) kg/m^2^). There were no significant differences in height, weight, age or BMI between the groups (*P*>0·05; Table 1). The mean caffeine intake for participants was 250 (sd 120) mg/d, and there was no difference in caffeine intake between the groups.

**Table 1:** Weight, BMI, and energy and sugar intakes at baseline and at the end of the study* (Mean values with their standard errors)

	Non-caffeinated SSB	Caffeinated SSB
	Baseline	End of the study	Baseline	End of the study
	Mean	se	Mean	se	Mean	se	Mean	se
Energy intake (kJ)	8555	398	8643	352	9077	452	9155	436
Sugar intake (g)	100	6	119	8	101	7	126	9
Weight (kg)	65·1	2	65·4	2	66·2	2	67·2	2
BMI (kg/m^2^)	22·9	0·4	23·0	0·4	23·1	0·5	23·3	0·5

SSB, sugar-sweetened beverage.

* There were no significant differences between the groups.

### Sugar-sweetened beverage consumption

**Fig. 2:**
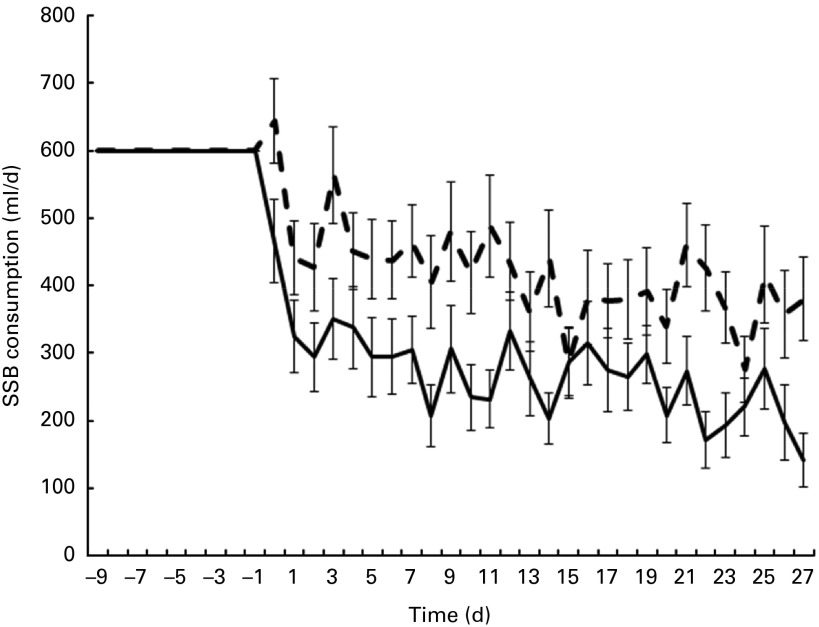
Sugar-sweetened beverage (SSB) consumption. From days − 9 to − 1 intake was maintained at 600 ml/d as a flavour-learning period. On days 0 to 27 consumption was *ad libitum*. Values are means, with standard errors represented by vertical bars. (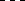), Caffeinated SSB consumption; (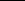), non-caffeinated SSB consumption.

A two-way between-group ANOVA was conducted to explore the influence of caffeine level and time on the consumption of the SSB. There was a statistically significant main effect for caffeine level (*F*(1, 2716) = 93·8, *P*< 0·001) and time (*F*(27, 2716) = 2·4, *P*< 0·001) (Fig. 2). There was no significant interaction between caffeine level and time (*F*(27, 2716) = 0·57, *P*= 0·964). There was a no correlation between liking of the SSB and consumption during the intervention period (*r* 0·024, *P*= 0·74).

The average daily consumption of the NC-SSB during the 28 d intervention was 273 (sd 278) ml, which was significantly lower than the daily consumption of 419 (sd 298) ml by the C-SSB group (Fig. 2). This translates to 512 (sd 521) and 785 (sd 559) kJ/d of energy from the SSB for the NC-SSB and C-SSB groups, respectively, with an average difference between the groups of 143 (sd 55) ml SSB/d (268 (sd 103) kJ/d) over the 28 d intervention period.

Participants were asked to only consume the study SSB during the intervention period; however, participants in the non-caffeinated group consumed an additional 135 (sd 30) ml/d (206 (sd 42) kJ/d) of non-study SSB and energy drinks, and participants in the C-SSB group consumed an additional 124 (sd 27) ml/d (192 (sd 41) kJ/d) of non-study SSB and energy drinks. Participants in the C-SSB group consumed 46 mg caffeine/d from the SSB, while all the participants continued their regular consumption of coffee and tea during the intervention period (250 (sd 130) mg/d). There were no significant differences between the two groups with respect to non-study SSB, energy drink, or caffeine consumption (*P*>0·05).

### Liking of sugar-sweetened beverages

A two-way between-group ANOVA was conducted to explore the influence of caffeine level and the beginning and end of the intervention on liking of the SSB. There was a statistically significant main effect for caffeine level (*F*(1, 194) = 5·3, *P*= 0·022), with the C-SSB being more liked than the NC-SSB (6·3 *v.* 6·0) (Table 2). There was no main effect for time (*F*(1, 198) = 2·4, *P*= 0·14) or interaction effect (*F*(1, 197) = 0·12, *P*= 0·73).

**Table 2:** Liking of the caffeinated and non-caffeinated sugar-sweetened beverages (SSB) at week 0 and week 6*

	Liking† (week 0)	Liking (week 6)
Caffeinated SSB	6·4	6·3
Non-caffeinated SSB	6·2	5·8

* There were no significant differences in the liking of SSB between the groups or over time.

† Nine-point hedonic scale was used to determine liking: 1, extremely dislike; 5, neither like nor dislike; 9, extremely like.

### Diet diary and anthropometry

There were no significant differences from baseline to day 28 in relation to energy intake, sugar intake or body weight between the groups (Table 1). There were no significant differences in caffeine intake between the groups (*P*>0·05).

### Ability to identify caffeine in the sugar-sweetened beverages

Of the participants, 67 % in the NC-SSB group correctly guessed their drink was non-caffeinated, while 51 % in the C-SSB group correctly guessed their SSB was caffeinated. There was no significant difference between the groups (*P*>0·05).

### Flavour difference between the sugar-sweetened beverages

There was no perceivable difference in flavour between the C-SSB and NC-SSB (*P*>0·05) as determined by trained tasting panel using the tetrad discrimination task. Any difference in liking or consumption between the SSB was not due to any difference in flavour.

## Discussion

The addition of 0·57 mm-caffeine to a lemon-flavoured SSB did not alter the flavour of the SSB, but was responsible for significantly increasing consumption in comparison to the NC-SSB. Therefore, caffeine as an additive in SSB has the ability to increase the consumption of the food in a free-living population.

### Caffeine as a flavouring

The addition of 0·57 mm-caffeine to the SSB had no flavour activity as trained tasters were unable to identify a difference between the C-SSB and NC-SSB. This supports earlier research suggesting that the concentration of caffeine is below flavour detection thresholds in common cola beverages^(^ ^10^ ^,^ ^12^ ^)^, and challenges the industry perspective that caffeine is a flavouring in cola SSB^(^ ^9^ ^)^. The C-SSB was more liked in the present study than the NC-SSB, with a major difference in liking observed at the end of the intervention. Overall, the flavour and liking data collected in the present study support previous speculation that the negative effects of caffeine withdrawal encourage repeat consumption of caffeine-containing beverages, rather than caffeine having any direct effects on the perceived flavour of SSB^(^ ^13^ ^,^ ^24^ ^)^.

### Caffeine as a driver of consumption

Caffeine, at concentrations found commonly in SSB, acts to increase consumption, presumably through subconscious positive effects as there was no difference in flavour or liking between the C-SSB and NC-SSB. Associative evidence indicated that caffeine has a role in consumption, with caffeine-containing beverages such as coffee, tea and cola SSB being the most highly consumed beverages worldwide^(^ ^1^ ^)^. We now have empirical data showing that caffeine, at concentrations commonly found in SSB, increases consumption. The time taken for caffeine to increase consumption occurred within the 9 d flavour-conditioning period when participants were asked to consume 600 ml of SSB leading into the *ad libitum* intake period^(^ ^18^ ^,^ ^25^ ^)^. At day 1 of the intervention period, participants in the caffeinated group were already consuming significantly more than the non-caffeinated group, and this difference was maintained over the 28 d intervention period.

### Caffeine in the obesogenic environment

Over time, the observed increased consumption of the C-SSB may be a significant contributor to the observed body-weight gain in the regular consumers of SSB^(^ ^2^ ^–^ ^4^ ^,^ ^7^ ^)^. The present study recruited regular SSB consumers who routinely consumed both C-SSB and NC-SSB. Therefore, even in a sample population already familiar with post-ingestive consequences, adding caffeine resulted in increased SSB consumption. Allowing caffeine to be added with little regulation to a growing number of products^(^ ^23^ ^)^ appears a recipe for overconsumption.

### Public health and policy implications

There are many factors that combine to promote the overconsumption of SSB including aggressive marketing, low satiation effect and decreased ability to compensate for liquid energy. In addition to these factors, the inclusion of caffeine as an additive in the formulation of these beverages may be considered a strategy to enhance the consumption of these products. As SSB are highly consumed, therefore, relatively small changes in composition have the potential to have significant benefits, reducing the development of overweight and obesity. We have previously calculated that removing caffeine from SSB, along with 10·3 % of sugar, has the potential to reduce body weight of adults by 600 g, without any change in SSB consumption^(^ ^8^ ^)^. The present study adds to the ‘caffeine–energy effect’ as we report that caffeine also increases the consumption of SSB. Taken together, caffeine as an additive in SSB has a significant influence on energy intake.

The results of the present study must be discussed in the broader context with a view to some limitations and future directions. The present study was not designed to determine any changes in weight or BMI as a 28 d intervention plus 9 d flavour conditioning was not a sufficient period of time for a significant weight change. Additionally, the study was not sufficiently powered to observe significant differences in energy intake between the groups. Future studies may wish to extend the intervention period to assess weight gain over a period longer than 28 d.

In summary, caffeine increases the consumption of SSB in comparison to a flavour-equivalent NC-SSB. As SSB are associated with the development of overweight and obesity, regulators and health professionals should increase the pressure on food companies to remove caffeine from formulations.
